# Nasal microbial composition and chronic otitis media with effusion: A case-control study

**DOI:** 10.1371/journal.pone.0212473

**Published:** 2019-02-22

**Authors:** Rebecca E. Walker, Caroline G. Walker, Carlos A. Camargo, Jim Bartley, David Flint, John M. D. Thompson, Edwin A. Mitchell

**Affiliations:** 1 Department of Paediatrics: Child and Youth Health, The University of Auckland, Auckland, New Zealand; 2 Centre for Longitudinal Research–He Ara ki Mua, Department of Population Health, The University of Auckland, Auckland, New Zealand; 3 Department of Emergency Medicine, Massachusetts General Hospital, Harvard Medical School, Boston, Massachusetts, United States of America; 4 Division of Otolaryngology-Head and Neck Surgery, Counties-Manukau District Health Board, Manukau SuperClinic, Manukau City, Auckland, New Zealand; University of Illinois at Urbana-Champaign, UNITED STATES

## Abstract

**Objectives:**

Chronic otitis media with effusion (COME) in children can cause prolonged hearing loss, which is associated with an increased risk of learning delays and behavioural problems. Dispersal of bacterial pathogens from the nasal passages to the middle ear is implicated in COME. We sought to determine whether there is an association between nasal microbial composition and COME in children.

**Methods:**

A case-control study of children aged 3 and 4 years was conducted. Cases undergoing placement of tympanostomy tubes for COME were compared to healthy controls. Nasal swabs were collected and a questionnaire was administered. The V1-3 region of the 16S rRNA gene was amplified, and sequenced on the Illumina MiSeq.

**Results:**

73 children with COME had a lower Shannon diversity index than 105 healthy controls (1.62 [.80] versus 1.88 [.84], respectively; *P* = .046). The nasal microbiota of cases and controls differed in composition using Bray-Curtis dissimilarity (p = 0.002). Children with COME had a higher abundance of otopathogens and lower abundance of commensals including alpha haemolytic *Streptococci* and *Lactococcus*. Cluster analysis revealed 4 distinct nasal microbial profiles. Profiles that were *Corynebacterium*-dominated (aOR 4.18 [95%CI, 1.68–10.39], *Streptococcus*-dominated (aOR 3.12 [95%CI, 1.08–9.06], or *Moraxella*-dominated (aOR 4.70 [95%CI, 1.73–12.80] were associated with COME, compared to a more mixed microbial profile when controlling for age, ethnicity, and recent antibiotics use.

**Conclusions:**

Children with COME have a less diverse nasal microbial composition with a higher abundance of pathogens, compared to healthy children who have a more mixed bacterial profile with a higher abundance of commensals. Further research is required to determine how nasal microbiota may relate to the pathogenesis or maintenance of COME, and whether modification of the nasal microbiota can prevent or treat children at risk of COME.

## Introduction

Chronic otitis media with effusion (COME) is diagnosed when fluid gathers behind the tympanic membrane for three months or longer [1]. The hearing loss associated with COME may impact language acquisition, and increase the risk of learning delays and behavioural problems [2].

COME has historically been regarded as a sterile inflammatory condition, as pathogens were seldom cultured from effusion in the middle ear (ME). However, with the advent of culture-independent methods, otopathogens have been found in COME as often as in the closely-related condition acute otitis media (AOM) [3, 4]. This discrepancy in findings may be explained by bacteria that are less likely to be detected by culture because they are residing in a biofilm state [5]. The key otopathogens are non-typeable *Haemophilus influenzae*, *Streptococcus pneumoniae*, *Moraxella catarrhalis*, and to a lesser extent *Staphylococcus aureus* [3].

While pathogenic bacterial biofilms residing in the ME may be involved in the maintenance of chronic effusion [6], it is unknown whether the nasal microbiota are also involved. The composition of both commensals and potential pathogens in the nasal microbiota may affect the risk of pathogens spreading to the ME. Commensal bacteria provide resistance against pathogen overgrowth by competing for nutrients and epithelial cell binding sites, producing antimicrobials, disrupting bacterial biofilms, and inducing the host’s immune system [7, 8].

Low bacterial diversity in a niche is often a marker of disease [9] and is associated with AOM [10]. Higher abundances of *Haemophilus*, *Moraxella*, and *Neisseria* in the nose have also been found to be associated with AOM [11]. Conversely, lower abundances of *Staphylococcus*, *Corynebacterium*, *Propionibacterium*, *Streptococcus* (usually not *S*. *pneumoniae*) and *Lactococcus* have been found in children with AOM [10, 11].

There are no published data on associations between nasal microbiota compositions and COME using cluster analysis. Nasal microbiota compositions characterized by *Moraxella*, *Streptococcus*, or *Haemophilus* have been reported to be associated with upper respiratory infection (URI) [12] asthma [13], AOM [12], pneumonia, or bronchiolitis [14, 15]. Protective compositions may also be emerging, with those dominated by *Staphylococcus*, *Sphingobium*, *Corynebacterium*, or *Dolosigranulum* associated with reduced risk of AOM [12] and asthma [16].

We investigated whether nasal microbial composition is associated with COME. Commensal bacteria in the nasal passages may protect against otopathogen overgrowth, or ongoing otopathogen overgrowth in the nasal passages may maintain persistence of effusion in the ME. We hypothesized that COME would be associated with a less diverse nasal microbiome; higher relative abundance of the otopathogens *H*. *influenzae*, *S*. *pneumoniae*, and *M*. *catarrhalis*; and lower relative abundance of the commensals *S*. *epidermidis*, *Corynebacterium*, *Propionibacterium*, *Dolosigranulum*, *Lactococcus*, *Lactobacillus*, and the alpha haemolytic *Streptococci* (AHS) spp.

## Methods

### Study design and setting

As previously reported, a case-control study of children aged 3 and 4 years was conducted in Auckland, New Zealand, to compare children with COME to healthy children [17]. Briefly, recruitment and data collection were conducted between May 2012 and November 2013. Cases were referred for tympanostomy tube placement (TTP), and had a recent medical history of COME and/or signs of COME confirmed by an otorhinolaryngologist during surgery. Controls were selected at random from children enrolled in primary care practices that had referred children for TTP in the previous year. Controls had no medical history of TTP, no episodes of otitis media with effusion (OME) lasting longer than one month in the past year, and never had OME lasting longer than three months. Cases and controls were excluded if they had craniofacial abnormalities or immunodeficiency.

### Ethics

The parent or legal guardian was informed about the study and provided written consent. The study was approved by the Northern X Regional Ethics Committee (reference NTX/11/EXP/027)

### Specimen collection

A sterile paediatric FLOQ swab (Copan, California, USA) moistened with sterile saline was inserted into subjects’ anterior nares. The swab was rotated in each nostril three times and placed into a 2ml tube of sterile STGG medium (skim milk, oxoid tryptone soya broth, glucose, and glycerol). Samples were stored at -80°C.

### DNA extraction, PCR, library preparation, sequencing, and bioinformatics

DNA was extracted from nasal samples and negative controls using the Qiagen Allprep kit (Qiagen, California, USA). We had 3 negative controls consisting of STGG broth and a swab exposed to the air, STGG broth only, and reagents only. The 16S ribosomal gene was PCR amplified using primers 27F and 534R to target the V1-3 region. Sequencing libraries were generated using the Nextera XT Index kit (Illumina Inc., San Diego. CA, USA) and amplicons sequenced on the Illumina MiSeq. USEARCH 64 (version 7) analysis pipeline was used. For operational taxonomic unit (OTU) assignment the UPARSE algorithm [18] was followed using a *de novo* picking approach with OTUs assignment clustered at 97% sequence similarity. Full details of the sample preparation, sequencing and nasal microbiota analysis are provided in Appendix A in S1 File.

Statistical analysis was carried out in R version 3.3.3 (www.r-project.org/) using the Phyloseq [19], vegan [20], DESeq2 [21], and cluster packages [22].

### Downstream analysis

OTUs were filtered to remove contaminants (see Appendix A in S1 File). OTUs with a total read number less than .005% of total reads and samples containing less than 1000 reads were removed. Additional species-level identification was achieved by aligning representative sequences using the Basic Local Alignment Search Tool (BLAST). Due to the limitations of classification using the 16S rRNA gene, these species identifications are not definitive.

### Subject characteristics

Statistical analysis was conducted using JMP 13, SAS Institute Inc., Cary, NC, 1989–2016. Categorical variables were analyzed using a chi-squared test and continuous variables were analyzed using a Student t-test. A *P* < .05 was considered statistically significant.

### Diversity analysis

Alpha diversity was calculated with absolute numbers using the Shannon diversity index in the R vegan package. Beta diversity was assessed with the Bray-Curtis dissimilarity measure. Permutation multivariate analysis of variance (PERMANOVA) using distance matrices was used to assess differences and significance was tested using ADONIS.

### Differential abundance

DESeq2 package from R was used for differential abundance analysis with Benjamini-Hochberg adjustment to control for multiple testing. We adjusted for age, ethnicity and antibiotic usage in the past month. A two-tailed *P* < .05 was considered statistically significant.

### OTU presence

For OTUs that were differentially abundant, presence of any reads was compared between cases and controls in JMP 13 using a Chi-square test with Benjamini-Hochberg adjustment, and a two-tailed *P* < .05 test for statistical significance.

### Cluster profiling

OTUs were collapsed at the genus level. To assess nasal microbiota profiles, we partitioned using a medoid clustering approach (PAM) and the Bray-Curtis dissimilarity metric. Number of clusters was selected using the gap statistic [23]. Using JMP 13, cluster was entered into a multivariable logistic regression model along with age, ethnicity, and antibiotics in the last month, which were identified as potential confounding variables. A two-tailed *P* < .05 test was considered statistically significant.

## Results

### Study population

Of 190 subjects who provided nasal samples, 12 were excluded due to having fewer than 1000 reads in total. The subjects whose samples were included did not differ from those whose samples were excluded, in terms of the subject characteristics listed in S1 Table.

As shown in Table 1, the mean age of the cases was younger than controls, there were more children of Asian ethnicity in the control group than in the case group, and the cases were younger than the controls when they were first given antibiotics. There was no difference between cases and controls for sex, season seen, method of delivery, being fully vaccinated, antibiotic exposure in the last month, attendance at daycare, or having older siblings.

**Table 1:** Characteristics of 178 children with chronic otitis media with effusion and healthy controls.

Variable	Cases *n* (%) n = 73	Controls *n* (%) n = 105	*P* value
Mean age in months [SD]	47.5 [6.7]	49.6 [6.8]	.05
Sex			.68
Female	29 (39.7)	45 (42.9)	
Male	44 (60.3)	60 (57.1)	
Ethnicity			.004
European & Other	45 (61.6)	51 (48.6)	
Asian	3 (4.1)	20 (19.1)	
Maori	14 (19.2)	19 (18.1)	
Pacific Island	11 (15.1)	15 (14.3)	
Season of sampling			.72
Summer (December-February)	12 (16.4)	14 (13.3)	
Autumn (March–May)	11 (15.1)	18 (17.1)	
Winter (June-August)	23 (31.5)	40 (38.1)	
Spring (September-November)	27 (37.0)	33 (31.4)	
Method of delivery at birth			.19
Normal vaginal delivery	41 (56.2)	58 (55.2)	
C-section–elective	6 (8.2)	17 (16.2)	
C-section–emergency	20 (27.4)	18 (17.1)	
Assisted vaginal delivery (forceps or ventouse)	6 (8.2)	12 (11.4)	
Fully vaccinated			.11
No	5 (6.9)	15 (14.4)	
Yes	68 (93.2)	89 (85.6)	
Mean age of first antibiotic (months) [SD]	10.9 [8.4]	18.8 [10.7]	< .001
Antibiotics in last month			.10
No	55 (75.3)	88 (87.1)	
Yes	11 (15.1)	6 (5.9)	
Unknown	7 (9.6)	7 (6.9)	
Mean daycare hours per week [SD]	24.2 [11.4]	21.2 [10.9]	.08
Older siblings			.65
No	31 (42.5)	41 (39.1)	
Yes	42 (57.5)	64 (61.0)	

### Microbiota analysis

A total of 3,606,750 sequences were assembled from pair-end reads across all samples. After filtering and chimera removal, 3,428,213 sequences were assembled into 300 OTUs. The median number of sequences per sample was 21,255 in cases (range 1,011–80,297) and 9,562 in controls (range 1,100–82,013).

OTU9 was classified in Greengenes as *Alloiococcus*, however the representative sequence matched *Dolosigranulum pigrum* in BLAST. This is a known misclassification in the Greengenes database. The representative sequence for OTU1 matched *Corynebacterium pseudodiptheriticum* using BLAST. OTU1 made up 97% of all reads of genus *Corynebacterium*.

### Core microbiome

The core nasal microbiome, defined as those OTUs present in over 80% of all samples, consisted of 16 taxa including *Corynebacterium*, *Moraxella*, *Streptococcus*, unknown *Bacilli* (*Staphylococcus*), *Alloiococcus/Dolosigranulum pigrum*, and *Neisseria*; see S2 Table.

### Diversity

**Fig 1:**
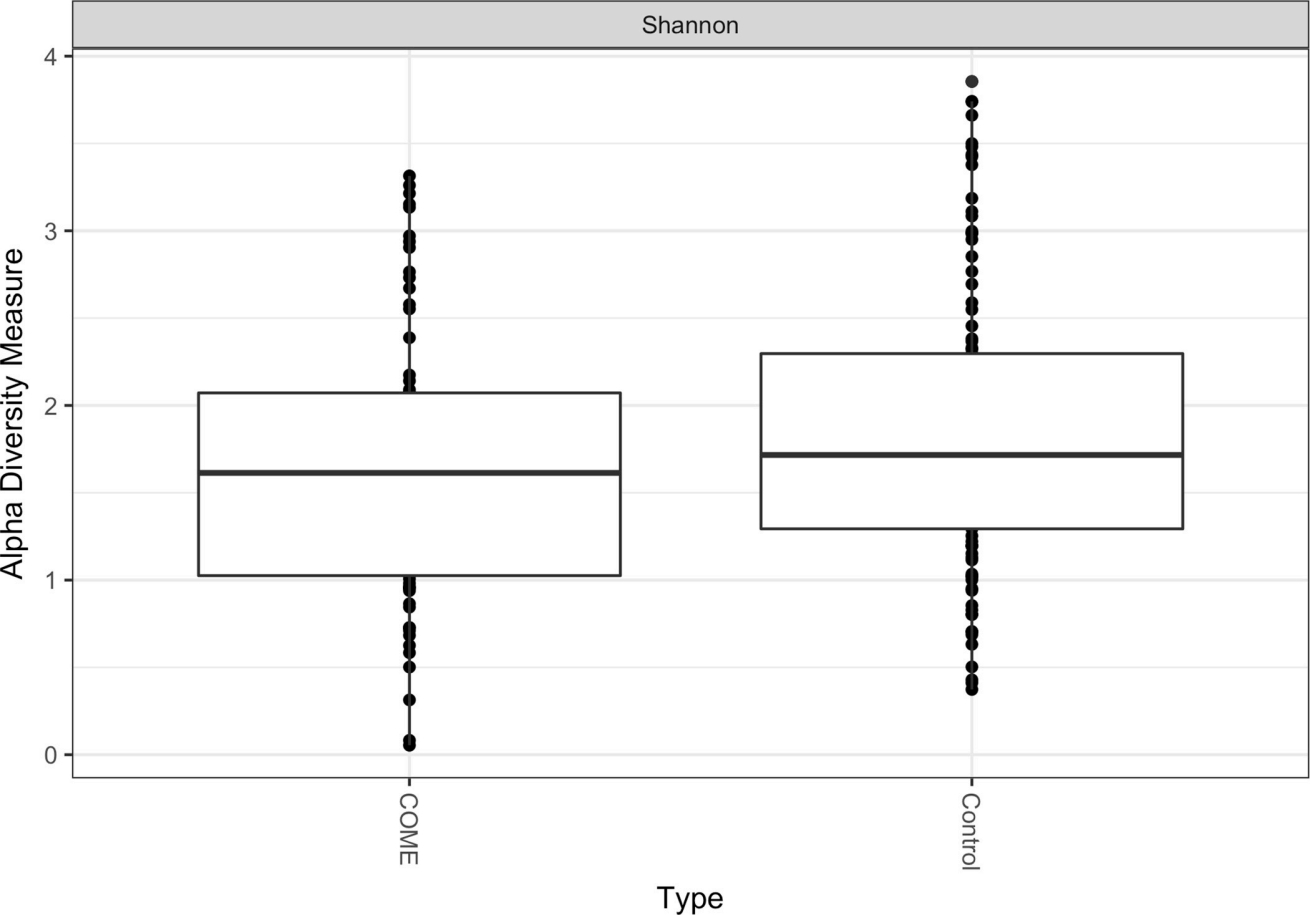
Shannon diversity index of the nasal microbiota of 73 children with chronic otitis media with effusion and 105 healthy controls. Diversity calculated using absolute abundance. Controls have higher alpha diversity than cases (*P* < .046).

Shannon diversity index was lower in children with COME than in healthy controls (mean = 1.62 [.80] versus mean = 1.88 [.84] respectively; *P* = .046); see Fig 1. Shannon diversity index was also measured using only subjects with the most frequently observed ethnicity (European) due to the strong inverse correlation between COME and Asian ethnicity. In this sensitivity analysis Shannon diversity was also lower in children with COME than in the healthy controls; see Appendix B in S1 File. We also found a significant difference in the microbial composition of children with COME and healthy controls (Bray-Curtis dissimilarity, unadjusted R2 = 0.02, *P* < .002); see S1 Fig.

### Differential abundance

**Fig 2:**
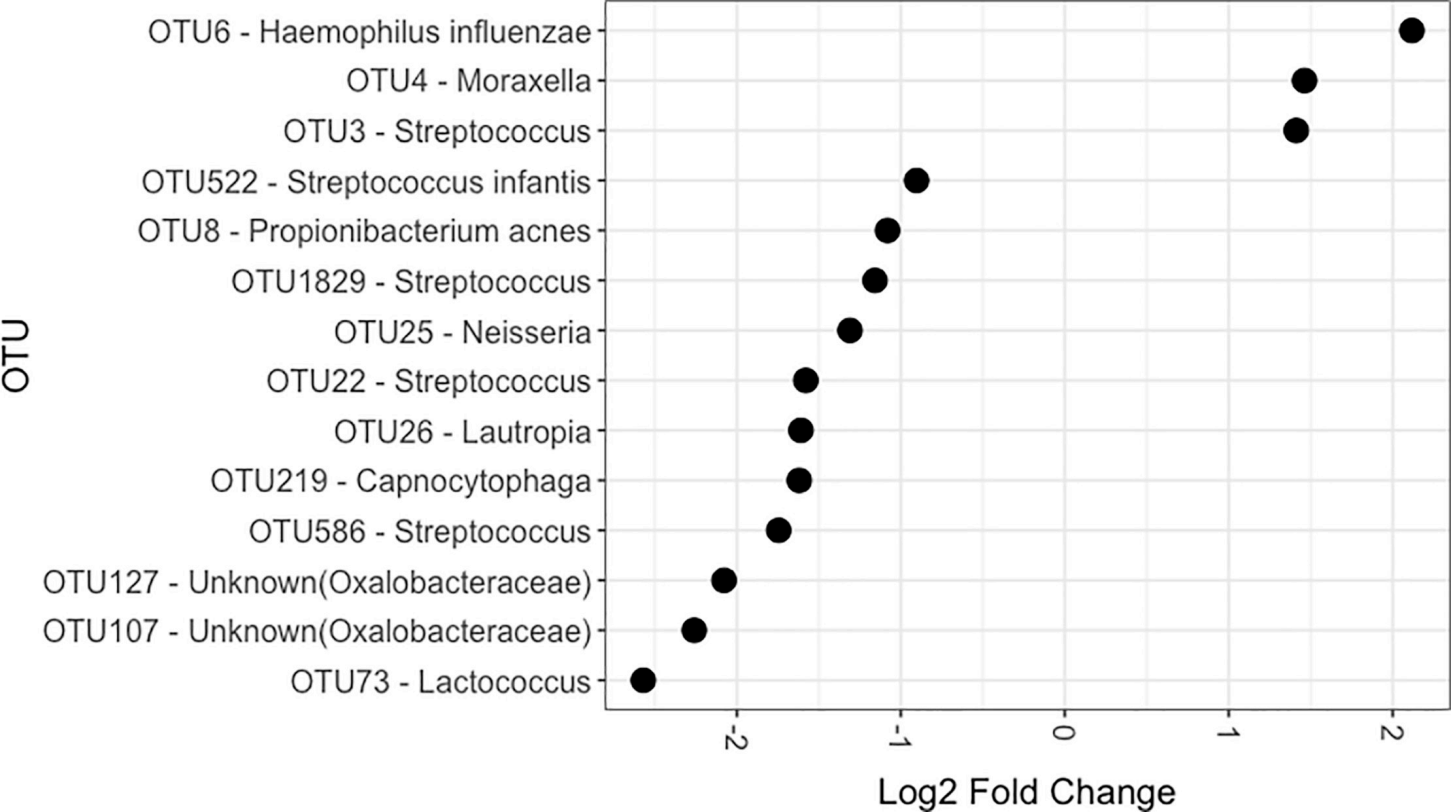
Differential abundance of the nasal microbiota. Calculated using R package DESeq2. Log2 fold change from 105 healthy controls to 73 children with chronic otitis media with effusion, controlling for age, ethnicity and recent antibiotic usage.

OTUs that were more common in children with COME were OTU3 (*Streptococcus)*, OTU4 (*Moraxella)*, and OTU6 (*H*. *influenzae)* (Fig 2). Examining their representative sequences on BLAST, OTU3 matched *S*. *pneumoniae (*but also *S*. *mitis* at 99%), and OTU4 matched *M*. *catarrhalis (*but also *M*. *caprae* at 99%).

All three OTUs that had greater abundance in the cases were also found to be present in a majority of the control samples (see S3 Table). OTU6 (*H*. *influenzae)* was found in 68% of the cases and 52% of the controls, OTU3 (*Streptococcus*) was found in 99% of the cases and 100% of the controls and OTU4 (*Moraxella*) was found in 92% of the cases and 85% of the controls. No significant differences were observed between detection of these OTUs in cases compared to controls.

Taxa more common in the healthy controls were three *Streptococcus* OTUs of uncertain species, *Streptococcus infantis*, *Propionibacterium acnes*, *Lactococcus* sp., *Neisseria* sp., *Lautropa* sp., *Capnocytophaga* sp., and two *Oxalobacteraceae* OTUs.

### Cluster profiles

**Fig 3:**
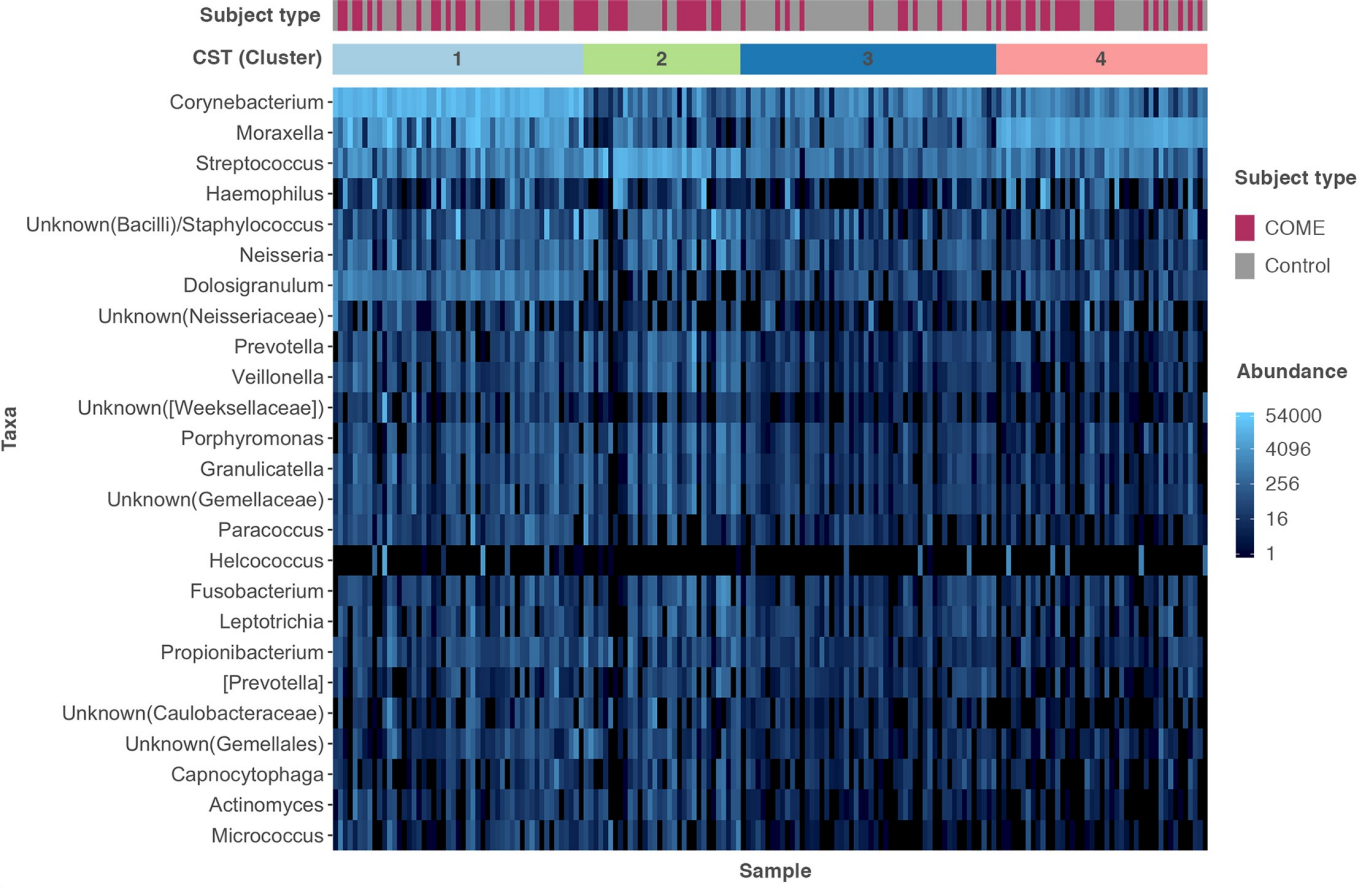
Cluster/Community structure type (CST) of the nasal microbiota of 73 children with chronic otitis media with effusion and 105 healthy controls. Profiles were partitioned using a medoid clustering approach (PAM) and the Bray-Curtis dissimilarity metric, based on absolute abundance. Four cluster profiles are indicated: *Corynebacterium*-dominated profile with prominent *Moraxella* and *Streptococcus* (1), *Streptococcus*-dominated profile (2), mixed profile (3), and *Moraxella*-dominated profile (4). A heatmap of the 25 most abundant genera is displayed adjacent.

Nasal microbiota clustered into 4 distinct profiles consisting of a *Corynebacterium*-dominated cluster (40% of case subjects were in this cluster, and 27% of control subjects), S*treptococcus*-dominated cluster (cases: 19%, controls: 15%), *Moraxella*-dominated cluster (cases: 27%, controls: 19%), and a mixed profile cluster (cases: 14%, controls: 39%); see Fig 3. Cluster was not associated with age, ethnicity, or antibiotics in the last month in univariable analyses. In an unadjusted analysis, *Corynebacterium*-dominated (OR 4.25 [95%CI, 1.84–10.47]), *Streptococcus*-dominated (OR 3.59 [95%CI, 1.34–9.98]), and *Moraxella*-dominated (OR 4.10 [95%CI, 1.64–10.72]) profiles were associated with COME, compared to the more mixed microbial profile. When controlling for potential confounders (age, ethnicity, and antibiotics in the last month), the *Corynebacterium*-dominated (aOR 4.18 [95%CI, 1.68–10.39], *Streptococcus*-dominated (aOR 3.12 [95%CI, 1.08–9.06]), and *Moraxella*-dominated (aOR 4.70 [95%CI, 1.73–12.80]) profiles remained associated with COME compared to the mixed profile.

## Discussion

Our findings that the nasal microbiota of children with COME had lower diversity, higher relative abundance of otopathogens, and a lower relative abundance of commensal bacteria–compared with healthy controls–indicates that the nasal microbiota may be important in initiating or maintaining COME.

Nasal and nasopharyngeal diversity have not previously been examined in OME or COME. Two studies have found low diversity to be associated with AOM [10, 24], two have found no association [11, 12], and one found that children with AOM had higher diversity [25]. The association observed between COME and low nasal diversity may reflect well-balanced microbiota exhibiting a higher resilience to infection, however the causal direction of such relationships has not been fully established [9]. It could equally signify that a bacterial infection that led to COME has also caused an overgrowth of pathogenic bacteria in the nose, which would result in lower alpha diversity by reducing the evenness of bacterial abundance.

A high load of pathogenic bacteria in the nasal passages may increase the risk of movement of those bacteria up the ET to the ME. *S*. *pneumoniae*, non-typeable *H*. *influenzae* and *M*. *catarrhalis* are the three main bacterial pathogens that have been identified in relation to COME [26], and are commonly detected in the ME effusion of children with COME [3]. Specific strains of these otopathogens are shared between the nasopharynx and the ME in individuals [27]. In some animal models, injection of bacteria into the nose results in ME carriage of those bacteria. [28] Polymicrobial infection further increases the risk of spread to the ME, as shown by nasal challenges using co-infection with multiple bacteria or respiratory viruses [29, 30].

We found that children with COME had higher nasal levels of *H*. *influenzae*, a *Streptococcus* OTU matching *S*. *pneumoniae* (and *S*. *mitis*), and a *Moraxella* OTU resembling *M*. *catarrhalis* (and *M*. *caprae*). *Haemophilus* has been found to be differentially abundant in children with recurrent AOM [25]. This correlation may reflect overgrowth of these pathogens initiating or maintaining COME by spreading to the ME. Although these three OTUs had higher differential abundance in the cases than the controls, they were all present in more than half of the controls. These OTUs may therefore be endogenous pathobionts, common microorganisms of children’s noses that may be disrupted and become infectious due to stimuli such URI [9]. URI often precedes OM, and can disrupt the airway microbiota. Viruses may promote adherence and virulence of otopathogens [31] by disrupting the airway epithelial barrier [32], decreasing mucociliary clearance [33], inducing the host to supply nutrients to pathogenic bacteria [34], and provoking biofilms to release virulent dispersed bacteria [35].

We also found that certain commensal bacteria were more common in controls. These included several AHS spp., a *Lactococcus* sp., and *Propionibacterium acnes*. AHS have been reported to help prevent otopathogen infection and OM [36]. Nasal *Lactococcus* also has higher relative abundance in healthy children than in children with AOM [11]. Propionibacteria appear to be protective against *S*. *pneumoniae* colonization, URI, and OM, and nasal levels are inversely associated with levels of *S*. *pneumoniae* and *S*. *aureus* [11, 37, 38].

We report the first cluster analysis of nasal bacterial profile in any OM-related condition. Our observation that a mixed nasal profile was inversely associated with COME compared with profiles dominated by *Corynebacterium*, S*treptococcus*, or *Moraxella* has not previously been reported in relation to OM. This finding may indicate that a more mixed profile is protective against infection. Adult profiles tend to be more mixed, more stable, and more resistant to infection that those of children [39].

The association between COME and a profile dominated by *Corynebacterium* was unexpected, as this genus has previously been inversely associated with otitis media [25]. Nearly all reads of *Corynebacterium* belonged to an OTU classified in BLAST as *Corynebacterium pseudodiptheriticum*. This species is an opportunistic pathogen [40, 41]. However, it was not differentially abundant in our cases. The association may therefore be due to a higher abundance of S*treptococcus* and *Moraxella* in the *Corynebacterium*-dominated cluster than in the mixed cluster, rather than a direct association with *Corynebacterium pseudodiptheriticum* itself.

Randomized controlled trials of probiotics have demonstrated that bacterial interference can be successful in reducing nasal colonization with otopathogens to treat URIs and OM in children [42], although not all trials show a protective effect for every condition [43]. Such studies support the plausibility of certain microbial compositions, such as the mixed bacterial profile that we observed, helping to protect against disease.

The study has potential limitations. Cases and controls differed by ethnicity, however similar results were found in a sensitivity analysis that examined all major findings in the largest single ethnicity, and when including ethnicity in a multivariable analysis. Limitations of 16S rRNA gene sequencing are that it is unable to distinguish between live and dead microbial DNA, and is poorly suited to distinguishing between many bacteria below the genus level, including genera that include both respiratory pathogens and commensals. We did identify the key otopathogen *H*. *influenzae* using 16S rRNA sequencing, however future research using whole genome sequencing would enable more precise differentiation between pathogen and commensal species and strains. We did not include analysis of the virome which may have provided additional clarification. Finally, this case-control study could not investigate the causal direction of the association between COME and nasal bacterial composition, therefore we recommend future longitudinal studies.

## Conclusion

The spread of pathogenic bacteria from the nasal passages to the ME may be involved in the pathogenesis of COME. Children with COME had a different nasal microbial profile to healthy controls, with lower diversity, higher abundance of pathogens, and lower abundance of commensals. Pathogens were equally likely to be present in the noses of healthy controls as in children with COME, and may therefore be endogenous pathobionts. Higher abundance of these pathobionts may increase the risk of COME, or help to maintain effusion once established. More research is required to determine the order of events leading to COME. If a more diverse and mixed nasal bacterial composition dominated by commensals proves to be protective against the spread of bacteria to the ME, this may present new opportunities to prevent or treat COME by influencing the microbiota of the nasal passages.

## Supporting information

S1 File Appendix A. Detailed Methods and Appendix B. Detailed Results.
(DOC)

S1 Table Comparison of characteristics of participants whose nasal samples were included in the analysis with those that had unusable samples, with Benjamini-Hochberg adjustment. (DOCX)

S2 Table The core microbiome of 73 children with chronic otitis media with effusion and 105 healthy controls. (DOCX)

S3 Table Presence of differentially abundant operational taxonomic units in 73 samples with chronic otitis media with effusion and 105 healthy controls, with Benjamini-Hochberg adjustment. (DOCX)

S1 Fig Non-metric multidimensional scaling (NMDS) ordination plot of Bray–Curtis community dissimilarities of OTUs for children with chronic otitis media with effusion and healthy controls, with variance stabilizing transformation to correct for difference in sequencing depth. (TIFF)
