# Supplementary material for: Nasal microbial composition and chronic otitis media with effusion: A case-control study
Source: PLoS One. 2019 Feb 22;14(2):e0212473. doi: 10.1371/journal.pone.0212473 (PMC6386383; doi:10.1371/journal.pone.0212473)
Supplement: S1 File — Appendix A. Detailed Methods and Appendix B. Detailed Results. (DOC) [file pone.0212473.s001.doc]

# Nasal microbial composition and chronic otitis media with effusion: A case-control study

Rebecca E. Walker^1*¶^, Caroline G Walker^2&^, Carlos A. Camargo Jr.^3¶^, Jim Bartley^4&^, David Flint^4^, John M. D. Thompson^1^, and Edwin A. Mitchell^1¶^

^1^Department of Paediatrics: Child and Youth Health, The University of Auckland, Auckland, New Zealand

^2^Centre for Longitudinal Research – He Ara ki Mua, Department of Population Health, The University of Auckland, Auckland, New Zealand

^3^Department of Emergency Medicine, Massachusetts General Hospital, Harvard Medical School, Boston, USA

^4^Division of Otolaryngology-Head and Neck Surgery, Counties-Manukau District Health Board, Manukau SuperClinic, Manukau City, Auckland, New Zealand

* Corresponding author

E-mail: [r.walker@auckland.ac.nz](mailto:r.walker@auckland.ac.nz) (REW)

¶These authors contributed equally to this work.

&These authors also contributed equally to this work.

**Appendix A.** Detailed Methods

**Appendix B.** Detailed Results

**Appendix A - Detailed Methods**

**DNA Extraction**

DNA was extracted from nasal samples and negative controls using the Qiagen Allprep kit (Qiagen, California, USA). Samples were centrifuged at full speed for 10 minutes to pellet microbial cells, the supernatant was discarded, and pellets were re-suspended in 700μL of buffer RLT Plus (including beta-mecaptoethanol). Samples were homogenised utilising 0.7mm garnet beads (Mobio, Carlsbad, USA) and a Tissuelyser II (Qiagen, California, USA) set for 4 minutes at 30Hz. Samples were then centrifuged to pellet cellular debris and the supernatant was used for DNA extraction according to the manufacturer’s instructions with one modification: at the DNA elution step the column was incubated at room temperature for 5 minutes before centrifuging to elute DNA from the column. All DNA samples were then purified using Agencourt Ampure beads (Beckman Coulter, California, USA). DNA purity was assessed using spectrophotometry (Nanodrop, ThermoFisher, Auckland, NZ) and DNA quantity was assessed using a Qubit (Life Technologies, Auckland, NZ).

**PCR and Sequencing**

The variable region of the 16S ribosomal gene was PCR amplified using primers 27F (5’- TCGTCGGCAGCGTCAGATGTGTATAAGAGACAG-3’) and 534R (5’- GTCTCGTGGGCTCGGAGATGTGTATAAGAGACAG-3’) to target the V1-3 region. The Roche Expand High Fidelity enzyme was used to amplify the target region using an Eppendorf thermal cycler. The PCR reaction volume was 25 μL, consisting of 0.5 μM of each primer, 0.5µL dNTPs (KAPA), 10% DMSO. Cycling conditions used were: [95°C for 10 minutes, (95°C for 10 seconds, 60°C for 30 seconds) × 50 cycles, 40°C for 40 seconds]. Amplicons were visualised on a 1.5% agarose gel to confirm correct product size. All amplicons were purified using Agencourt Ampure beads (Beckman Coulter, California, USA). Purity was assessed using spectrophotometry (Nanodrop) and quantity was determined using a Qubit (Life Technologies, Auckland, NZ). Equimolar dilutions of each sample was provided to the sequencing provider for indexing and sequencing.

**Library Preparation**

Two sequencing runs (96 samples per run) were performed in total utilising the MiSeq platform 600 cycle kit (300bp x2 paired-end) at the Centre for Genomics, Proteomics and Metabolomics (CGPM), The University of Auckland, Auckland, New Zealand. Amplicon cleaning, indexing, and sequencing were performed according to the Illumina MiSeq 16S Metagenomic Sequencing Library Preparation Protocol (Illumina, San Diego, Calif). Briefly, 5µL of the purified V1-V3 amplicon was used for the index PCR, followed by PCR purification using Agencourt Ampure beads (Beckman Coulter, California, USA). 5µL of each library (96 per run) was pooled, the pooled library was quantified using a Qubit (Life Technologies, Auckland, NZ) and an aliquot was validated using the Bioanalyzer DNA 1000 chip. The library denaturation reaction included a 20% PhiX spike in per run. The cases and controls were distributed evenly across the plates.

**Bioinformatic Processing**

USEARCH 64 (version 7) analysis pipeline was utilized. Forward and reverse reads were merged for each sample with reads of less than 250bp removed, a minimum merge length of 450, and a minimum overlap region of 50bp. Paired reads were filtered using a minimum expected quality filter of 1.0. Chimeras were removed using UCHIME. For operational taxonomic unit (OTU) assignment the UPARSE pipeline^26^ was followed using a *de novo* picking approach with OTUs assignment clustered at 97% sequence similarity. QIIME (version 1.9.1) was then used^27^ where taxonomy of OTUs was predicted using RDP classifier trained with the Greengenes dataset to build a phylogenetic tree. A mock community control and negative controls were included to assess the robustness of the analysis. Mock community results were found to correlate between sequencing runs with pairwise correlations > 0.95.

**Downstream Analysis**

Contaminants removed included Chloroplasts, mitochondria, Methylobacteriaceae, Sphingomonadacea*e*, *Geobacillus*, *Thermus*, *Anoxybacillus*, *Agrobacterium*, *Curvibacter* and *Brevundimonas* as these are known environmental contaminants^31^ that were found in our negative control samples. OTUs matching these taxonomies were removed from our analysis.

**Appendix B - Detailed Results**

**Sensitivity Analysis**

In the subgroup analysis of European children only (n= 45 cases and 47 controls), Shannon diversity was lower in children with COME (mean = 1.62 [0.87]) than in the healthy controls (mean = 2.01 [0.92]); *P*=.04.
