# Supplementary material for: Nasal microbial composition and chronic otitis media with effusion: A case-control study
Source: PLoS One. 2019 Feb 22;14(2):e0212473. doi: 10.1371/journal.pone.0212473 (PMC6386383; doi:10.1371/journal.pone.0212473)
Supplement: S1 Table — (DOCX) [file pone.0212473.s002.docx]

**S1 Table:** Comparison of characteristics of participants whose nasal samples were included in the analysis with those that had unusable samples, with Benjamini-Hochberg adjustment

| **Variable** | **Cases included in analysis *n* (%) n = 73** | **Cases not included in analysis *n* (%) n = 13** | ***P* value** | **Adjusted *P* value** | **Controls included in analysis *n* (%) n = 105** | **Controls not included in analysis *n* (%) n = 11** | ***P* value** | **Adjusted *P* value** |
| --- | --- | --- | --- | --- | --- | --- | --- | --- |
| Mean age in months [SD] | 47.5 [6.7] | 48.6 [6.3] | .57 | .81 | 49.6 [6.6] | 51.5 [5.9] | .34 | .80 |
| Sex |  |  | .93 | .93 |  |  | .68 | .85 |
| Female | 29 (39.7) | 5 (38.46) |  |  | 45 (42.86) | 4 (36.36) |  |  |
| Male | 44 (60.27) | 8 (61.54) |  |  | 60 (57.14) | 7 (63.64) |  |  |
| Ethnicity |  |  | .36 | .80 |  |  | .92 | .93 |
| European & Other | 45 (61.6) | 7 (53.9) |  |  | 51 (48.6) | 5 (45.5) |  |  |
| Asian | 3 (4.1) | 0 (0.0) |  |  | 20 (19.1) | 2 (18.2) |  |  |
| Maori | 14 (19.2) | 5 (38.5) |  |  | 19 (18.1) | 2 (18.2) |  |  |
| Pacific Island | 11 (15.1) | 1 (7.7) |  |  | 15 (14.3) | 2 (18.2) |  |  |
| Season of blood sampling |  |  | .55 | .81 |  |  | .79 | .93 |
| Summer (December-February) | 12 (16.4) | 1 (7.7) |  |  | 14 (13.3) | 1 (9.1) |  |  |
| Autumn (March–May) | 11 (15.1) | 4 (30.8) |  |  | 18 (17.1) | 2 (18.2) |  |  |
| Winter (June-August) | 23 (31.5) | 4 (30.8) |  |  | 40 (38.1) | 3 (27.3) |  |  |
| Spring (September-November) | 27 (37.0) | 4 (30.8) |  |  | 33 (31.4) | 5 (45.5) |  |  |
| Method of delivery |  |  | .50 | .81 |  |  | .26 | .80 |
| Unassisted | 41 (56.2) | 9 (69.2) |  |  | 58 (55.2) | 7 (63.6) |  |  |
| C-section – elective | 6 (8.2) | 0 (0.0) |  |  | 17 (16.2) | 0 (0.0) |  |  |
| C-section – emergency | 20 (27.4) | 3 (23.1) |  |  | 18 (17.1) | 3 (27.3) |  |  |
| Instruments | 6 (8.2) | 1 (7.7) |  |  | 12 (11.4) | 1 (9.1) |  |  |
| Fully vaccinated |  |  | .34 | .80 |  |  | .61 | .81 |
| No | 5 (6.9) | 2 (15.4) |  |  | 15 (14.4) | 1 (9.1) |  |  |
| Yes | 68 (93.2) | 11 (84.6) |  |  | 89 (85.6) | 10 (90.9) |  |  |
| Mean age of first antibiotic (months) [SD] | 10.9 [8.4] | 18.8 [10.7] | .03 | .30 | 18.8 [10.7] | 19.4 [9.3] | .87 | .93 |
| Antibiotics in last month |  |  | .33 | .80 |  |  | .46 | .81 |
| No | 55 (75.3) | 10 (83.3) |  |  | 88 (87.1) | 9 (90.0) |  |  |
| Yes | 11 (15.1) | 2 (16.7) |  |  | 6 (5.9) | 1 (10.0) |  |  |
| Unknown | 7 (9.6) | 0 (0.0) |  |  | 7 (6.9) | 0 (0.0) |  |  |
| Mean daycare hours per week [SD] | 24.2 [11.4] | 22.2 [10.6] | .54 | .81 | 21.2 [10.9] | 25.9 [13.8] | .29 | .80 |
| Older siblings |  |  | .17 | .80 |  |  | .03 | .30 |
| No | 31 (42.5) | 3 (23.1) |  |  | 41 (39.1) | 8 (72.7) |  |  |
| Yes | 42 (57.5) | 10 (76.9) |  |  | 64 (61.0) | 3 (27.3) |  |  |
