# Supplementary material for: Nasal microbial composition and chronic otitis media with effusion: A case-control study
Source: PLoS One. 2019 Feb 22;14(2):e0212473. doi: 10.1371/journal.pone.0212473 (PMC6386383; doi:10.1371/journal.pone.0212473)
Supplement: S2 Table — (DOCX) [file pone.0212473.s003.docx]

**Table S2:** The core microbiome of 73 children with chronic otitis media with effusion and 105 healthy controls. These OTUs were present in over 80% of all samples. Percentages are relative abundance of OTU reads in cases and controls.

| **OTU** | **Bacteria** | **Case (%)** | **Control (%)** |
| --- | --- | --- | --- |
| OTU1 | *Corynebacterium* | 30.5 | 32.4 |
| OTU4 | *Moraxella* | 13.9 | 10.6 |
| OTU3 | *Streptococcus* | 11.9 | 12.1 |
| OTU5 | *Unknown Bacilli (S. aureus)* | 8.5 | 3.1 |
| OTU9 | *Alloiococcus/* *Dolosigranulum pigrum* | 1.8 | 2.0 |
| OTU12 | *Neisseria subflava* | 1.3 | 1.6 |
| OTU8 | *Propionibacterium acnes* | .4 | .6 |
| OTU522 | *Streptococcus infantis* | .5 | .9 |
| OTU25 | *Neisseria* | .3 | .7 |
| OTU19 | *Unknown Gemellaceae* | .6 | .8 |
| OTU31 | *Veillonella* | .6 | .5 |
| OTU45 | *Unknown Gemellales* | .4 | .4 |
| OTU28 | *Granulicatella* | .3 | .4 |
| OTU22 | *Streptococcus* | .3 | .9 |
| OTU586 | *Streptococcus* | .2 | 1.1 |
| OTU68 | *Streptococcus* | .3 | .4 |
