# Supplementary material for: Nasal microbial composition and chronic otitis media with effusion: A case-control study
Source: PLoS One. 2019 Feb 22;14(2):e0212473. doi: 10.1371/journal.pone.0212473 (PMC6386383; doi:10.1371/journal.pone.0212473)
Supplement: S3 Table — (DOCX) [file pone.0212473.s004.docx]

**Table S2:** Presence of differentially abundant operational taxonomic units in samples with chronic otitis media with effusion and healthy controls, with Benjamini-Hochberg adjustment

| **OTU** | **Bacteria** | **Case (%)** | **Control (%)** | ***P* value** | **Adjusted *P* value** |
| --- | --- | --- | --- | --- | --- |
| OTU3 | *Streptococcus (S. pneumoniae/mitis group)* | 98.7 | 100 | .18 | .23 |
| OTU4 | *Moraxella (M. catarrhalis)* | 91.8 | 85.7 | .21 | .24 |
| OTU6 | *Haemophilus influenzae* | 68.5 | 52.4 | .03 | .05 |
| OTU73 | *Lactococcus (L. lactis)* | 27.4 | 58.1 | <.001 | .001 |
| OTU586 | *Streptococcus (mitis group)* | 75.3 | 87.6 | .04 | .05 |
| OTU26 | *Lautropia* | 54.8 | 74.3 | .007 | .02 |
| OTU22 | *Streptococcus (S. thermophilus)* | 78.1 | 91.4 | .01 | .02 |
| OTU25 | *Neisseria* | 75.3 | 89.5 | .01 | .02 |
| OTU219 | *Capnocytophaga* | 27.0 | 49.0 | .004 | .02 |
| OTU1829 | *Streptococcus (S. sanguinis)* | 71.2 | 75.2 | .55 | .55 |
| OTU522 | *Streptococcus infantis* | 93.2 | 90.4 | .40 | .43 |
| OTU8 | *Propionibacterium acnes* | 84.9 | 95.2 | .02 | .03 |
| OTU107 | *Unknown(Oxalobacteraceae)* | 27.4 | 52.4 | <.001 | .006 |
| OTU127 | *Unknown(Oxalobacteraceae)* | 24.7 | 43.8 | .008 | .02 |
