# Supplementary figures and images for: Nasal microbial composition and chronic otitis media with effusion: A case-control study
Source: PLoS One. 2019 Feb 22;14(2):e0212473. doi: 10.1371/journal.pone.0212473 (PMC6386383; doi:10.1371/journal.pone.0212473)

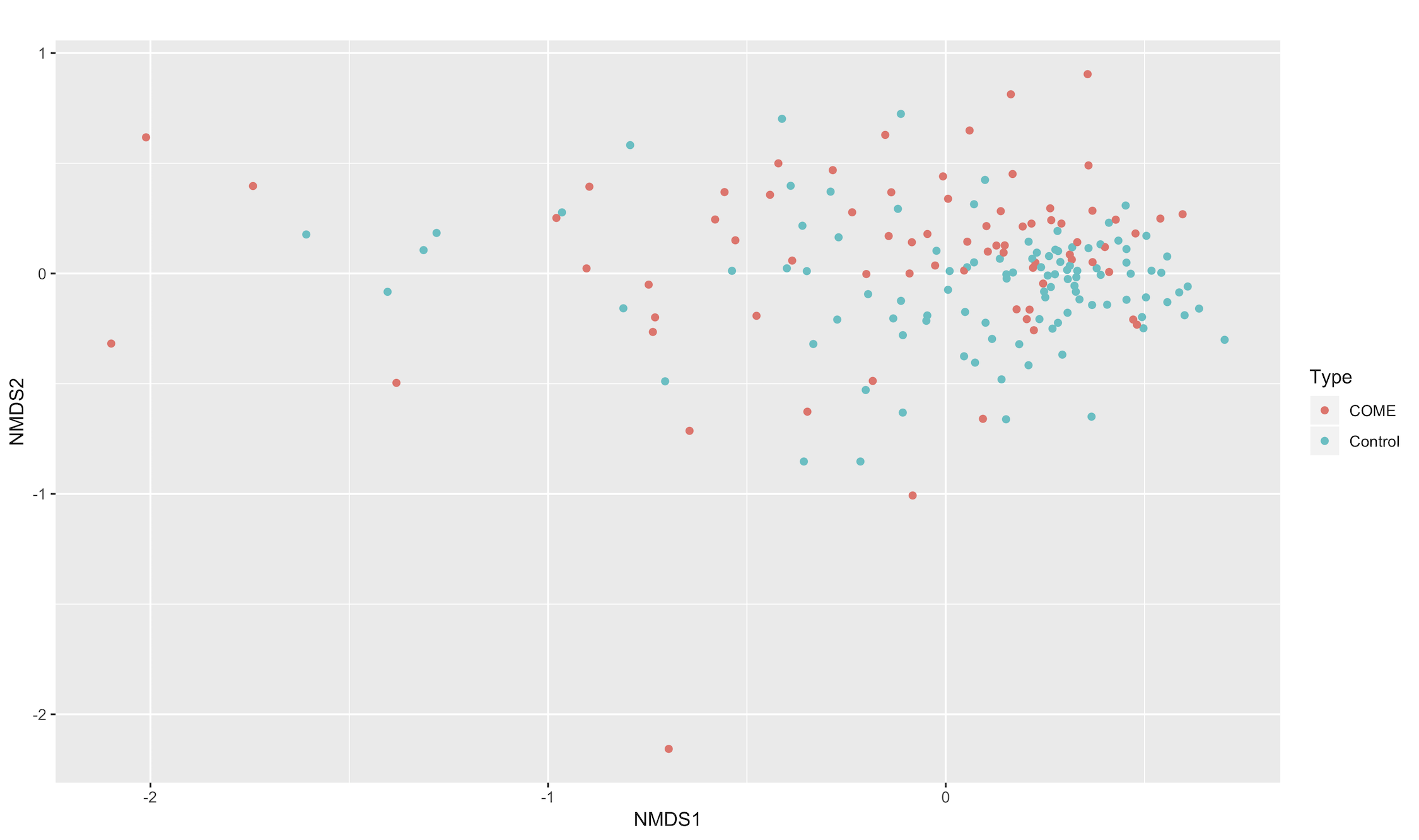

Supplement: S1 Fig — (TIFF) [file pone.0212473.s005.tiff]
